# Invasive Blood Pressure Measurement and In-hospital Mortality in Critically Ill Patients With Hypertension

**DOI:** 10.3389/fcvm.2021.720605

**Published:** 2021-09-01

**Authors:** Bin Zhou, Liang-Ying Lin, Xiao-Ai Liu, Ye-Sheng Ling, Yuan-Yuan Zhang, An-Qi Luo, Meng-Chun Wu, Ruo-Mi Guo, Hua-Li Chen, Qi Guo

**Affiliations:** ^1^Department of Cardiology, The Third Affiliated Hospital of Sun Yat-sen University, Guangzhou, China; ^2^Institute of Nursing, Guangdong Food and Drug Vocational College, Guangzhou, China; ^3^Department of Nuclear Medicine, National Cancer Center/National Clinical Research Center for Cancer/Cancer Hospital, Chinese Academy of Medical Sciences and Peking Union Medical College, Beijing, China; ^4^Department of Emergency Medicine, Sun Yat-sen Memorial Hospital of Sun Yat-sen University, Guangzhou, China; ^5^Department of Radiology, The Third Affiliated Hospital of Sun Yat-sen University, Guangzhou, China; ^6^Department of Nosocomial Infection Control, The Third Affiliated Hospital of Sun Yat-sen University, Guangzhou, China; ^7^Department of Cardiology, Sun Yat-sen Memorial Hospital of Sun Yat-sen University, Guangzhou, China

**Keywords:** invasive blood pressure, hypertension, hospital mortality, intensive care unit, propensity score

## Abstract

**Background:** Invasive blood pressure (IBP) measurement is common in the intensive care unit, although its association with in-hospital mortality in critically ill patients with hypertension is poorly understood.

**Methods and Results:** A total of 11,732 critically ill patients with hypertension from the eICU-Collaborative Research Database (eICU-CRD) were enrolled. Patients were divided into 2 groups according to whether they received IBP. The primary outcome in this study was in-hospital mortality. Propensity score matching (PSM) and inverse probability of treatment weighing (IPTW) models were used to balance the confounding covariates. Multivariable logistic regression was used to evaluate the association between IBP measurement and hospital mortality. The IBP group had a higher in-hospital mortality rate than the no IBP group in the primary cohort [238 (8.7%) vs. 581 (6.5%), *p* < 0.001]. In the PSM cohort, the IBP group had a lower in-hospital mortality rate than the no IBP group [187 (8.0%) vs. 241 (10.3%), *p* = 0.006]. IBP measurement was associated with lower in-hospital mortality in the PSM cohort (odds ratio, 0.73, 95% confidence interval, 0.59–0.92) and in the IPTW cohort (odds ratio, 0.81, 95% confidence interval, 0.67–0.99). Sensitivity analyses showed similar results in the subgroups with high body mass index and no sepsis.

**Conclusions:** In conclusion, IBP measurement was associated with lower in-hospital mortality in critically ill patients with hypertension, highlighting the importance of IBP measurement in the intensive care unit.

## Introduction

Hypertension is a prevalent condition and is a major contributing risk factor for numerous diseases, such as heart failure, myocardial infarction, stroke, and chronic kidney disease (1, 2). The diagnosis and management of hypertension depend on the accurate and rapid measurement of blood pressure (BP) (3, 4). The direct measurement of BP requires invasive BP (IBP) measurement, although IBP is not practical in most cases. The development of devices has led to the availability of several non-invasive means of measuring BP that have been found to have acceptable accuracy when standardized techniques and appropriate observer training are implemented (5, 6). Thus, non-invasive BP measurements are widely used in clinical, ambulatory, home, and hospital settings (7).

For critically ill patients with hypertension in the intensive care unit (ICU), an effective and efficient BP measurement method is needed to support clinicians making critical clinical decisions. Therefore, critically ill patients with hemodynamic instability undergo IBP measurement (8). Moreover, clinically significant discrepancies have been observed between invasive and non-invasive systolic BP measurements in patients with hypotension in the ICU, supporting the importance of IBP measurement in this setting (9). However, there is little direct evidence available regarding whether IBP measurement could help achieve a better prognosis in critically ill patients with hypertension.

In this study, clinical data from critically ill patients with hypertension in the eICU-Collaborative Research Database (eICU-CRD) were enrolled. Propensity score matching was used to balance the potential confounding covariates. The objective of this study was to evaluate the association between IBP measurement and in-hospital mortality in critically ill patients with hypertension and provide evidence of the utility of IBP measurement in the ICU.

## Materials and Methods

### Data Source

Cohort data were extracted from the eICU-CRD, which contains records for 139,367 patients and 200,859 total ICU admissions across the United States collected from 2014 to 2015. The database has been made available by Philips Healthcare in partnership with the Computational Physiology Laboratory at the Massachusetts Institute of Technology (10). We used the eICU-CRD database version 2.0, which is publicly available through the PhysioNet website (https://physionet.org/content/eicu-crd/2.0/). The data include hourly physiological readings from bedside monitors, records of demographic characteristics, diagnoses according to the ninth revision of the International Classification of Diseases (ICD-9) codes, and other clinical data collected during routine medical care. The eICU-CRD database has been approved by the institutional review board of the Massachusetts Institute of Technology and certified by the US Health Insurance Portability and Accountability Act (No. 1031219-2).

### Study Population

Structured Query Language with PostgreSQL (version 9.6) was used to extract the data from the eICU-CRD database. Hypertensive patients were enrolled. The inclusion criteria were as follows: (1) a diagnosis of hypertension according to the ICD-9 codes and an age >18 years old; (2) the first ICU admission, if multiple ICU admissions occurred for the same patient; and (3) a stay in the ICU longer than 1 day. The exclusion criteria were as follows: (1) patients with no non-invasive BP within the first 24 h after ICU admission; (2) patients with no Acute Physiology and Chronic Health Evaluation (APACHE IV) score.

### IBP and In-hospital Mortality

We set the searching timeline as <24 h before ICU admission or during ICU stay. The patients who had IBP records during this searching timeline were categorized as the IBP group, with the remaining patients were included in the no IBP group. The primary outcome of the study was in-hospital mortality during each patient's first hospital admission.

### Covariates

Baseline characteristics within the first 24 h after ICU admission were collected, including age, sex, and body mass index (BMI). The APACHE IV score and vital signs, including mean heart rate, mean non-invasive systolic BP, mean non-invasive diastolic BP, use of mechanical ventilation, use of vasopressors, and use of sedative drugs during the first 24 h after ICU admission, were extracted. To better show hemodynamic status, the lowest BP during the ICU stay was also extracted. The first value in the initial 24 h after ICU admission for each laboratory test was used, including the white blood cell count, hemoglobin level, platelet count, blood urea nitrogen (BUN) level, creatinine level, glucose level, bicarbonate level, potassium level, and sodium level. Comorbidities identified by ICD-9 codes were also extracted, including coronary artery disease, atrial fibrillation, congestive heart failure, diabetes mellitus, chronic obstructive pulmonary disease (COPD), renal disease, cancer, sepsis, and shock. For each variable, the missing value ratio was <10%. Missing values were imputed based on the random forest model using the missForest package (11).

### Statistical Analysis

The full set of original participants constituted the primary cohort. In addition to the primary cohort, propensity score matching was used to assemble well-balanced groups, namely, the propensity-score matched (PSM) cohort. The propensity score was estimated using a non-parsimonious multivariable logistic regression model, with the IBP measurement as the dependent variable and all of the baseline characteristics as the independent variables. Patients who underwent IBP measurement were matched 1:1 to patients who did not undergo IBP measurement according to the propensity score using the greedy nearest neighbor matching algorithm with a caliper width of 0.2. In addition, an inverse probability of treatment weighing (IPTW) cohort was created using the estimated propensity scores as weights.

Values are presented as the means (standard deviations) or medians (interquartile ranges) for continuous variables, and categorical variables are presented as numbers (percentages). The standardized mean differences (SMDs) were calculated to evaluate the effectiveness of the propensity score matching with regard to balancing the IBP group and the no IBP group (12). The chi-square test was used to evaluate the difference in in-hospital mortality between the IBP and no IBP groups. The aim of this study was to evaluate the association between IBP measurement and in-hospital mortality in critically ill hypertension patients. And logistic regression analysis was the statistical technique widely used to predict the relationship between the dependent outcome and the independent variable. Thus, logistic regression was enrolled in this current study. Logistic regression was then performed with the primary cohort, PSM cohort and IPTW cohort separately. The model was adjusted for a series of variables that were considered clinically relevant or differed between IBP group and no IBP group, including age, sex, BMI, APACHE IV score, non-invasive systolic BP, non-invasive diastolic BP, use of ventilation, use of sedatives, use of vasopressors, glucose level, sodium level, platelet count, potassium level, BUN level, creatinine level, coronary heart disease, congestive heart failure, atrial fibrillation, COPD, renal disease, and sepsis. Sensitivity analyses were conducted to evaluate the robustness of the findings of the study in subgroups stratified by the presence of atrial fibrillation, the presence of sepsis, the APACHE IV score, the BMI, and the lowest BP.

All statistical analyses were performed using R (version 4.0.1, R Foundation for Statistical Computing, Vienna, Austria), and *p* < 0.05 was considered statistically significant.

## Results

**Figure 1:**
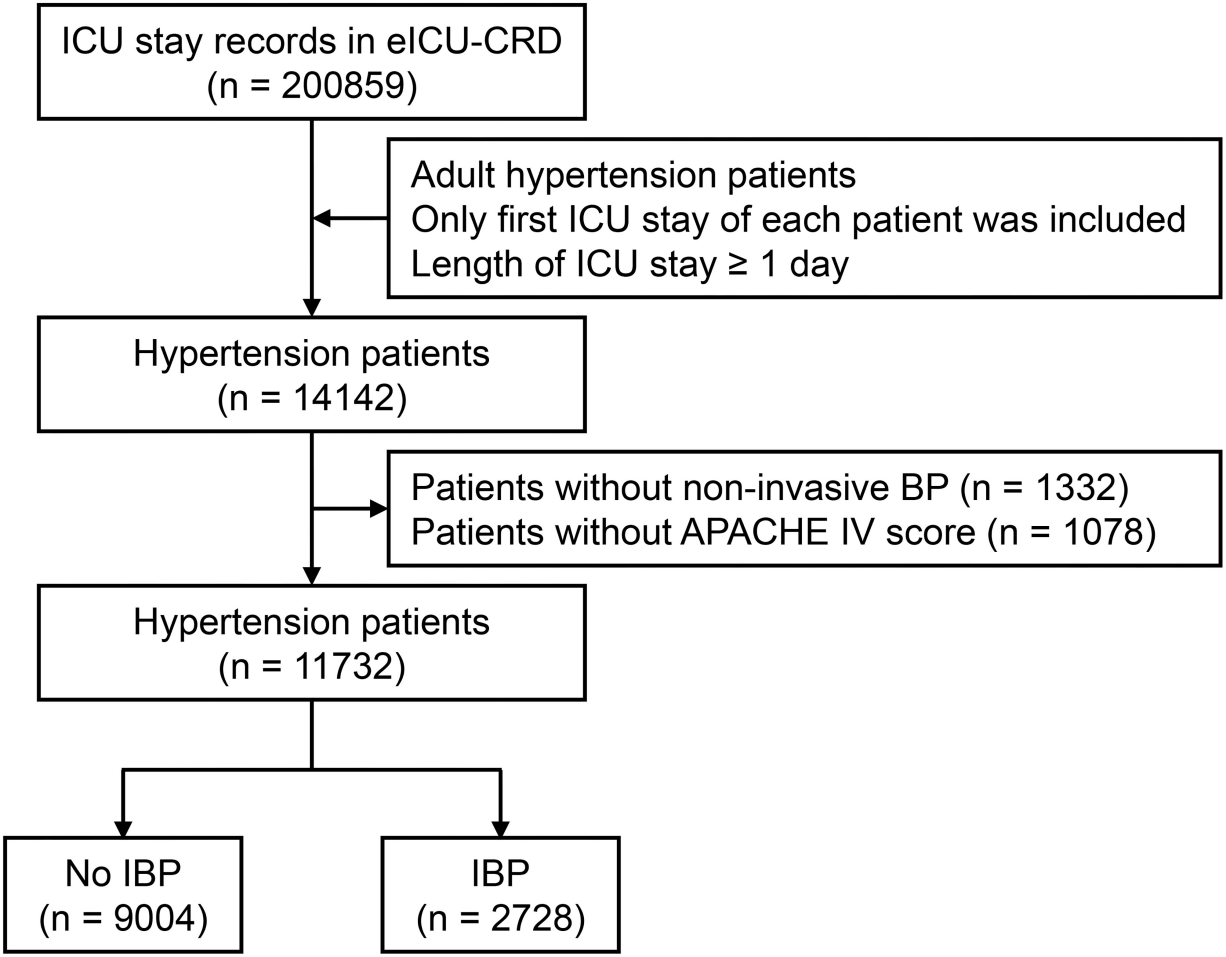
Flow chart of subject enrollment. A total of 11,732 hypertensive patients from eICU-CRD were enrolled in this study. ICU, intensive care unit; eICU-CRD, eICU Collaborative Research Database; BP, blood pressure; APACHE, Acute Physiology and Chronic Health Evaluation; IBP, invasive blood pressure.

**Figure 2:**
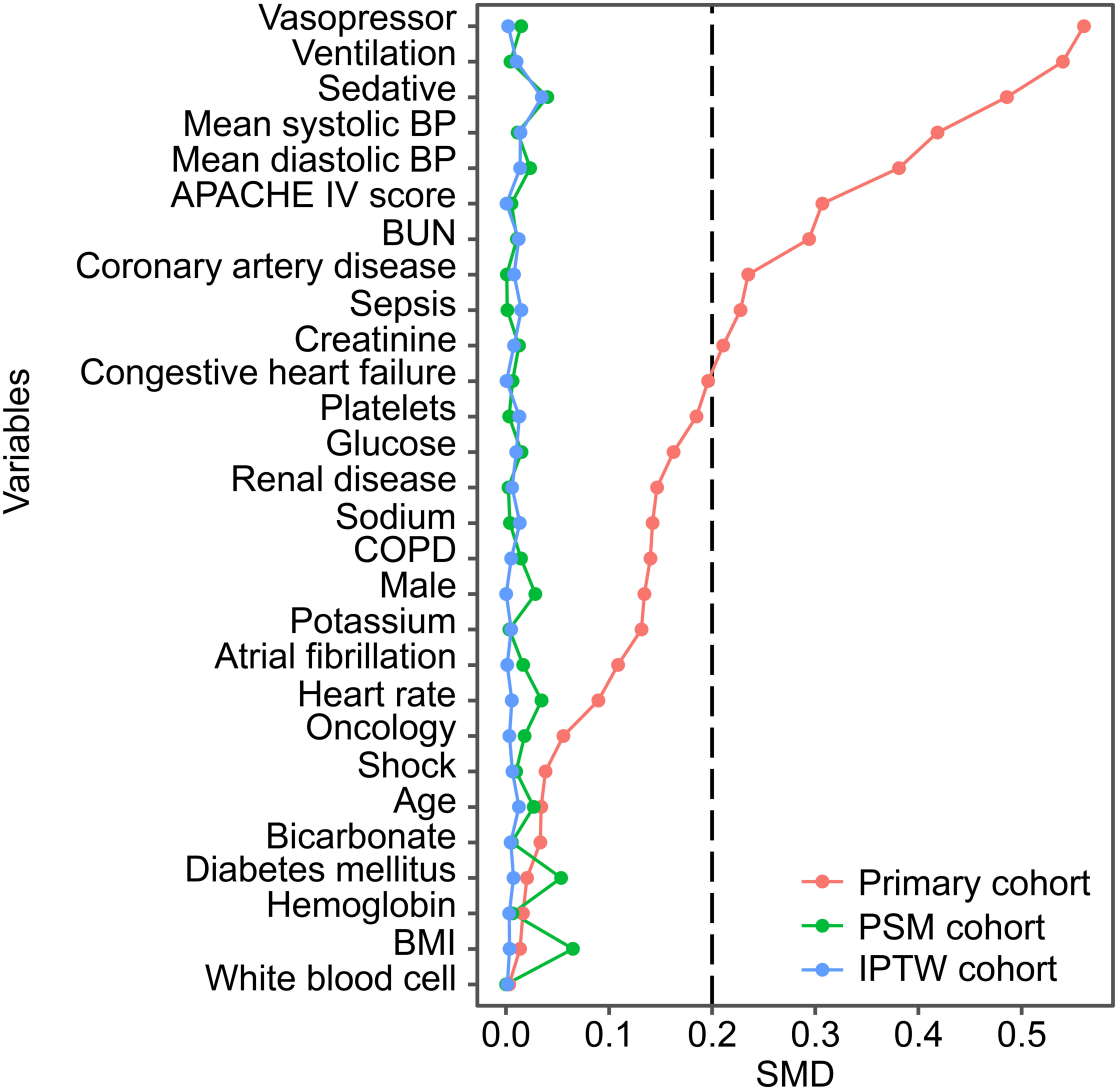
SMD between the no IBP and IBP groups in each cohort. Variables were ranked by the SMD. BP, blood pressure; APACHE, Acute Physiology and Chronic Health Evaluation; BUN, blood urea nitrogen; COPD, chronic obstructive pulmonary disease; BMI, body mass index; PSM, propensity-score matched; IPTW, inverse probability of treatment weighting; SMD, standardized mean difference.

A total of 11,732 hypertensive patients were enrolled in this current study, with 9,004 patients in the no IBP group and 2,728 patients in the IBP group (Figure 1). The mean age of the study patients was 65.37 ± 14.29 years old, and 6,372 (54.3%) were males. In total, 819 (7.0%) patients died in the hospital. After propensity score matching, 2,342 patients in the no IBP group and 2,342 patients in the IBP group were enrolled in the PSM cohort (Table 1). Before matching, the majority of the variables were not balanced between the 2 groups, except for age, heart rate, white blood cell count, bicarbonate level, hemoglobin level, and presence of cancer, shock, and diabetes mellitus. The unbalanced covariates were balanced after matching in the PSM cohort and IPTW cohort (Figure 2).

**Table 1:** Baseline characteristics of subjects in the primary and PSM cohorts.

	**Primary cohort**			**PSM cohort**		
	**No IBP**	**IBP**	**SMD**	**No IBP**	**IBP**	**SMD**
Number	9,004	2,728		2,342	2,342	
Age, years	65.26 ± 14.70	65.73 ± 12.84	0.034	65.71 ± 14.38	65.34 ± 12.94	0.027
Male	4,751 (52.8)	1,621 (59.4)	0.134	1,367 (58.4)	1,400 (59.8)	0.029
BMI, kg/m^2^	30.22 ± 10.08	30.39 ± 13.77	0.014	30.29 ± 10.07	31.11 ± 14.61	0.065
Heart rate, /min	83.56 ± 16.15	82.18 ± 14.41	0.090	81.55 ± 15.81	82.08 ± 14.41	0.035
Mean systolic BP, mmHg	133.51 ± 20.90	124.63 ± 21.51	0.419	126.34 ± 19.04	126.58 ± 21.71	0.011
Mean diastolic BP, mmHg	70.98 ± 13.67	65.97 ± 12.62	0.381	66.72 ± 12.24	67.02 ± 12.75	0.024
Lowest systolic BP, mmHg	96.20 ± 20.86	91.63 ± 20.10	0.224	90.04 ± 18.98	93.28 ± 19.93	0.166
Lowest diastolic BP, mmHg	47.31 ± 14.17	45.76 ± 12.76	0.115	43.80 ± 12.79	46.68 ± 12.65	0.227
Vasopressor use	535 (5.9)	699 (25.6)	0.561	384 (16.4)	397 (17.0)	0.015
Sedative use	2,436 (27.1)	1,365 (50.0)	0.486	1,104 (47.1)	1,057 (45.1)	0.040
Ventilation use	1,520 (16.9)	1,104 (40.5)	0.540	805 (34.4)	800 (34.2)	0.004
APACHE IV score	51.00 (38.00–64.00)	55.00 (41.00–75.00)	0.307	54.00 (41.00–72.00)	53.00 (40.00–71.00)	0.005
White blood cells, × 10^9^/L	10.99 ± 5.82	10.97 ± 5.41	0.003	10.94 ± 5.79	10.94 ± 5.34	<0.001
Hemoglobin, g/dl	12.25 ± 2.50	12.29 ± 2.31	0.017	12.37 ± 2.48	12.36 ± 2.32	0.006
Platelets, × 10^9^/L	222.00 (173.00–276.00)	206.00 (162.00–258.00)	0.185	209.00 (162.00–260.00)	208.00 (164.00–260.00)	0.003
BUN, mg/dL	21.00 (14.00–33.00)	18.00 (13.00–25.00)	0.294	19.00 (13.00–27.00)	18.00 (13.00–26.00)	0.011
Creatinine, mg/dL	1.10 (0.83–1.73)	1.02 (0.80–1.40)	0.211	1.03 (0.80–1.41)	1.02 (0.81–1.40)	0.013
Glucose, mg/dL	141.00 (112.00–198.00)	142.00 (116.00–181.25)	0.163	140.00 (111.00–188.00)	144.00 (117.00–185.00)	0.015
Bicarbonate, mmol/L	24.93 ± 5.25	24.77 ± 4.30	0.034	24.93 ± 4.95	24.90 ± 4.31	0.006
Potassium, mmol/L	4.18 ± 0.79	4.08 ± 0.65	0.132	4.09 ± 0.70	4.08 ± 0.66	0.003
Sodium, mmol/L	137.24 ± 5.47	137.95 ± 4.38	0.142	137.83 ± 4.88	137.85 ± 4.38	0.004
Coronary artery disease	1,730 (19.2)	797 (29.2)	0.235	647 (27.6)	646 (27.6)	0.001
Atrial fibrillation	1,246 (13.8)	281 (10.3)	0.109	237 (10.1)	249 (10.6)	0.017
Congestive heart failure	1,656 (18.4)	312 (11.4)	0.196	280 (12.0)	275 (11.7)	0.007
Diabetes mellitus	2,855 (31.7)	839 (30.8)	0.021	695 (29.7)	753 (32.2)	0.054
COPD	1,392 (15.5)	293 (10.7)	0.140	273 (11.7)	262 (11.2)	0.015
Renal disease	1,582 (17.6)	337 (12.4)	0.147	298 (12.7)	296 (12.6)	0.003
Cancer	523 (5.8)	196 (7.2)	0.056	174 (7.4)	163 (7.0)	0.018
Sepsis	1,471 (16.3)	241 (8.8)	0.228	224 (9.6)	223 (9.5)	0.001
Shock	1,219 (13.5)	406 (14.9)	0.039	320 (13.7)	312 (13.3)	0.010

*Data are presented as the means ± standard deviations, medians (interquartile ranges), or numbers (percentages) as appropriate. PSM, propensity-score matched; IBP, invasive blood pressure; SMD, standardized mean difference; BMI, body mass index; BP, blood pressure; APACHE, Acute Physiology and Chronic Health Evaluation; BUN, blood urea nitrogen; COPD, chronic obstructive pulmonary disease*.

**Figure 3:**
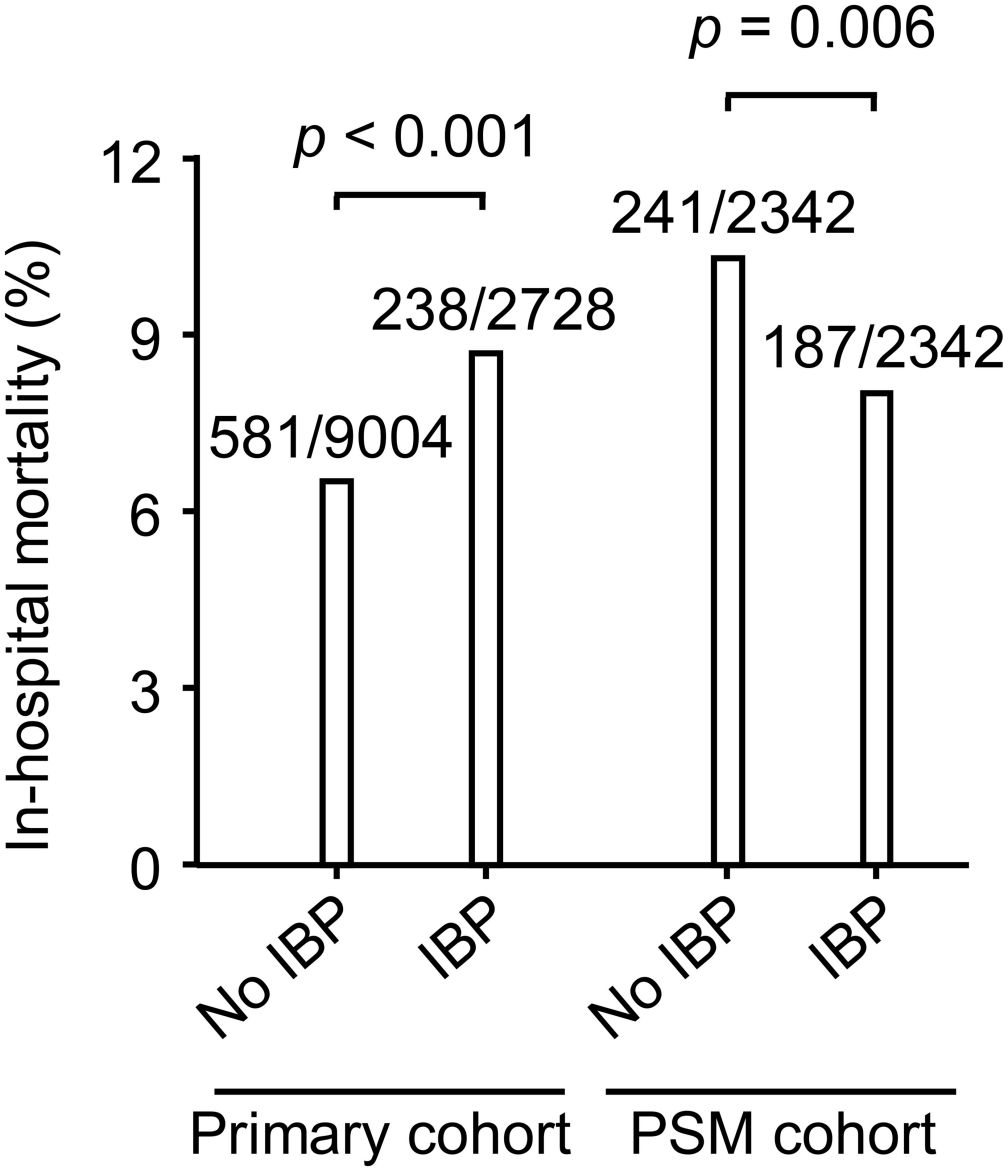
In-hospital mortality in the primary cohort and PSM cohort. Differences in in-hospital mortality were evaluated using the chi-square test. Data were shown as death events/total patients. PSM, propensity-score matched; IBP, invasive blood pressure.

**Figure 4:**
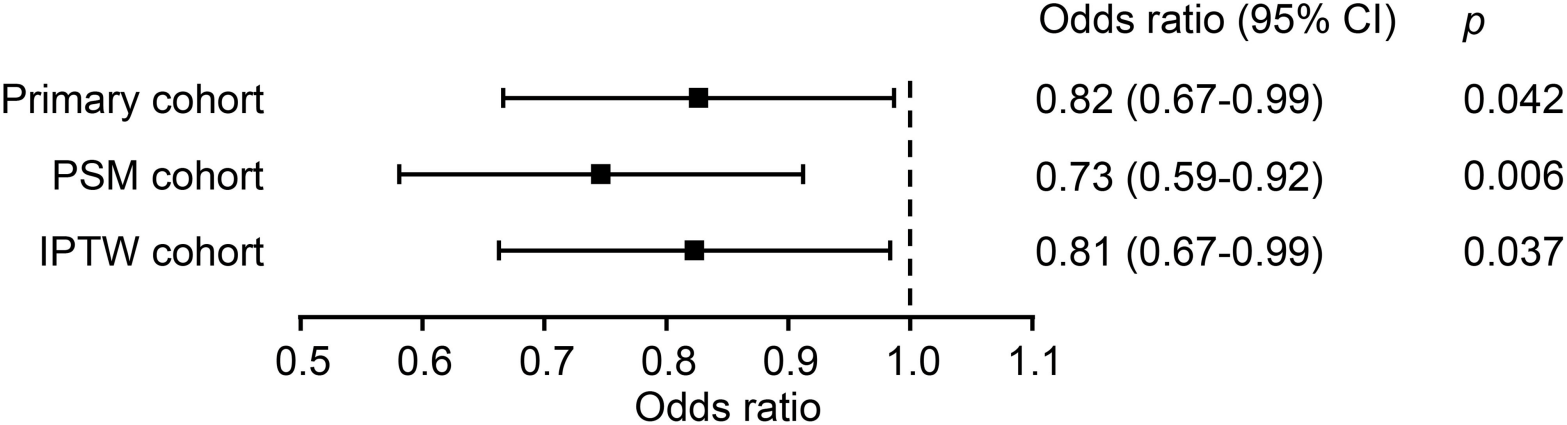
Association between IBP measurement and in-hospital mortality in each cohort. PSM, propensity-score matched; IPTW, inverse probability of treatment weighting; CI, confidence interval.

The chi-square test showed that the IBP group had a higher in-hospital mortality rate than the no IBP group in the primary cohort [238 (8.7%) vs. 581 (6.5%), *p* < 0.001]. In the PSM cohort, the IBP group had a lower in-hospital mortality rate than the no IBP group [187 (8.0%) vs. 241 (10.3%), *p* = 0.006] (Figure 3). After adjustment, logistic regression in the primary cohort showed that IBP measurement was associated with a lower hospital mortality rate, with an odds ratio of 0.82 (95% CI, 0.67–0.99). This association remained significant in the PSM cohort (odds ratio, 0.73, 95% CI, 0.59–0.92) and in the IPTW cohort (odds ratio, 0.81, 95% CI, 0.67–0.99) (Figure 4).

The possible interactive effects of IBP measurement and the included variables on in-hospital mortality were evaluated. An interactive effect was observed between IBP measurement and sepsis (*p* for interaction = 0.049). The association between IBP measurement and in-hospital mortality remained significant in the subgroup without sepsis (odds ratio, 0.69, 95% CI, 0.53–0.88) but not in the subgroup with sepsis (odds ratio, 1.00, 95% CI, 0.60–1.68). No significant interactive effects were observed between IBP measurement and atrial fibrillation, the APACHE IV score, or BMI (*p* for interaction > 0.05) (Table 2).

**Table 2:** Sensitivity analyses of the association between IBP measurement and in-hospital mortality in the PSM cohort.

**Subgroup**	**Number of patients**	**Number of death events**	**Odds ratio (95% CI)**	***p***	***p* for interaction**
Atrial fibrillation					0.765
Yes	486	75	0.75 (0.41–1.36)	0.345	
No	4,198	353	0.72 (0.57–0.92)	0.007	
Sepsis					0.049
Yes	447	104	1.00 (0.60–1.68)	0.994	
No	4,237	324	0.69 (0.53–0.88)	0.003	
APACHE IV score					0.157
> 57	2,016	348	0.81 (0.63–1.04)	0.106	
≤ 57	2,668	80	0.44 (0.27–0.71)	0.001	
BMI					0.316
≥ 28	2,680	231	0.66 (0.49–0.89)	0.007	
<28	2,004	197	0.89 (0.63–1.24)	0.489	
Lowest systolic BP <90 mmHg or					0.008
Lowest diastolic BP <60 mmHg					
Yes	4,204	403	0.70 (0.55–0.87)	0.002	
No	480	25	1.04 (0.30–3.85)	0.952	

*IBP, invasive blood pressure; PSM, propensity-score matched; CI, confidence interval; APACHE, Acute Physiology and Chronic Health Evaluation; BMI, body mass index*.

## Discussion

IBP measurement is common in the ICU, although its association with in-hospital mortality has been poorly investigated. In this study, propensity score matching, multivariable logistical regression, and sensitivity analyses were performed. Our study found that IBP measurement was associated with a lower in-hospital mortality rate in critically ill patients with hypertension in the ICU, demonstrating the utility of IBP measurement in clinical practice.

The accurate measurement of BP is essential for the diagnosis and management of hypertension. To date, the dynamic monitoring of BP in hospital settings has depended on ambulatory blood pressure monitoring, which is a non-invasive and fully automated technique (13). Recently, it was reported that non-invasive cuff BP failed to identify 28% of isolated systolic hypertension cases, suggesting the need to improve cuff BP measurements (14). In ICUs, it was also shown that non-invasive BP measurement significantly underestimated BP compared with IBP measurement (15). Moreover, differences between non-invasive and IBP measurements have been shown to trigger low-risk treatment decisions in 20% of the patients in the ICU (16). These studies showed the importance of IBP measurement for achieving a diagnosis and making decisions, while the association between IBP measurement and mortality in critically ill patients with hypertension remains poorly understood. IBP is generally preferred over non-invasive blood pressure recordings when critical decisions need to be made regarding critically ill patients with hemodynamic instability (8). Our study showed that critically ill hypertension patients who underwent IBP measurement had a lower in-hospital mortality rate than those who did not undergo IBP measurement. These results highlight the essential role of IBP measurement in critically ill patients with hypertension, and the use of IBP measurement should not be omitted.

More than half of the variables showed marked differences between the IBP and no IBP groups based on the baseline characteristics and SMD plot. The IBP group had higher proportions of patients using vasopressors, ventilation, and sedatives and a higher APACHE IV score. Given the fact that the IBP group was more severely ill, it is unsurprising that the IBP group had a higher in-hospital mortality rate than the no IBP group. Indeed, substantial heterogeneity in baseline characteristics in critically ill patients is common, and randomized controlled trials are rarely feasible in the ICU (17, 18). The propensity score matching method balances the distributions of confounding covariates (19). Since its development, it has been used in a wide array of studies on cardiovascular disease (20, 21). In this study, the propensity score matching method was used to address the imbalances in baseline characteristics between the 2 groups. Interestingly, the in-hospital mortality in the IBP group was significantly lower than that in the no IBP group in the PSM cohort. After adjusting for a series of confounding covariates, IBP measurement was shown to be significantly associated with a lower in-hospital mortality rate in the primary cohort, PSM cohort and IPTW cohort. These consistent results provide direct evidence supporting the benefits associated with the use of IBP measurement in critically ill patients with hypertension.

The current common method of non-invasive BP measurement relies on an arm cuff and the application of the intermittent automated oscillometric technique (22). Arm size and conicity were found to influence the accuracy of non-invasive BP and to be associated with BMI (23, 24). According to the subgroup analyses in patients with different BMIs, IBP measurement was association with a better prognosis in critically hypertensive patients with high BMI values, although no significant interactive effect was observed. BP monitoring is crucial for achieving the target BP range in sepsis patients (25). Recently, central venous pressure measurement was reported to be associated with decreased 28-day mortality in sepsis patients (26). In our study, a significant interactive effect was observed between sepsis and IBP measurement on in-hospital mortality. IBP measurement was shown to be useful in critically ill hypertensive patients without sepsis. Nevertheless, sepsis was demonstrated suffer obvious heterogeneity in critically ill patients (27, 28). Different sepsis subtypes might respond differently to the IBP monitoring, which is worthy of further investigating.

Although our findings are important, several limitations should be considered. First, due to the retrospective study design, selection bias could not be avoided. The propensity score matching method and sensitivity analyses were performed to guarantee the robustness of our results. Second, the identification of hypertension was based on the ICD-9 codes but not clinical diagnostic criteria; thus, a few patients might have been missed. Third, we excluded the patients without APACHE II score instead of imputation. This exclusion might influence the underlying association between IBP and mortality. In addition, some other variables, including lactate, which played important roles in the severity of illness were also not available due to the excessive missing value. Fourth, in the sensitivity analyses, the statistical power might have been insufficient due to the small sample sizes in some subgroups, such as in the sepsis subgroup.

In conclusion, IBP measurement was associated with a lower in-hospital mortality rate in critically ill patients with hypertension, highlighting the importance of IBP measurement in the ICU.

## Data Availability Statement

Publicly available datasets were analyzed in this study. This data can be found at: https://physionet.org/content/eicu-crd/2.0.

## Ethics Statement

The studies involving human participants were reviewed and approved by the institutional review board of the Massachusetts Institute of Technology. The patients/participants provided their written informed consent to participate in this study.

## Author Contributions

QG, H-LC, and R-MG conceived and designed the research protocol. BZ, L-YL, and X-AL collected and analyzed the data and wrote the first draft of the manuscript. All authors provided input on data analysis, interpretations and participated in multiple revisions of the manuscript, approved the final version of the manuscript, and agree to be accountable for all aspects of the work.

## Conflict of Interest

The authors declare that the research was conducted in the absence of any commercial or financial relationships that could be construed as a potential conflict of interest.

## Publisher's Note

All claims expressed in this article are solely those of the authors and do not necessarily represent those of their affiliated organizations, or those of the publisher, the editors and the reviewers. Any product that may be evaluated in this article, or claim that may be made by its manufacturer, is not guaranteed or endorsed by the publisher.
